# Driving HIA implementation in Ireland: The role of academia and public health institutions

**DOI:** 10.1093/eurpub/ckac129.100

**Published:** 2022-10-25

**Authors:** M O'Mullane

**Affiliations:** Institute for Social Science in the 21st Century, University College Cork, Cork, Ireland

## Abstract

Over the past decades public health research has presented compelling evidence that health is socially determined. To address structural inequalities and inequities in health, public policies require intersectoral development and implementation. Health Impact Assessment (HIA) is an established tool for analysing potentially detrimental health outcomes of policies, programmes and projects. In Ireland, we are presented with a unique opportunity to examine HIA implementation by capitalising on the synergy of recent policy commitments driving HIA implementation. Specifically, the national public health framework Healthy Ireland 2013-2025, and the newly developed all-island Institute of Public Health HIA guidance (2021), have coalesced in steering the strategic direction of HIA. This presentation outlines the current synergistic policy context on the island of Ireland, including the publication of new HIA guidance, which opens opportunities and new possibilities for implementing HIA. Reflections will be made on the potential and reality of current HIA training programmes in Ireland. Learning from the lessons of a dearth of HIA implementation at varying time periods since 2000, this presentation discusses the mechanisms in place, such as the successful World Health organization-led healthy cities projects in Ireland, such as Cork Healthy Cities, and those key drivers that are required to capture policy synergy for meaningful HIA implementation.

